# In Vitro Evaluation of the Accuracy of Three Electronic Apex Locators Using Different Sodium Hypochlorite Concentrations

**DOI:** 10.3390/medicina59050918

**Published:** 2023-05-11

**Authors:** Sanda Ileana Cîmpean, Radu Marcel Chisnoiu, Adela Loredana Colceriu Burtea, Rareș Rotaru, Marius Gheorghe Bud, Ada Gabriela Delean, Ioana-Sofia Pop-Ciutrilă

**Affiliations:** 1 Department of Odontology, Endodontics and Oral Pathology, “Iuliu Hațieganu” University of Medicine and Pharmacy, 33 Moților Street, 400001 Cluj-Napoca, Romania; sanda.cimpean@umfcluj.ro (S.I.C.); loredana.colceriu@umfcluj.ro (A.L.C.B.); marius.bud@umfcluj.ro (M.G.B.); ada.delean@umfcluj.ro (A.G.D.); ioanasofia_ciutrila@yahoo.com (I.-S.P.-C.); 2 Student at Faculty of Dental Medicine, “Iuliu Hațieganu” University of Medicine and Pharmacy, 400001 Cluj-Napoca, Romania; rares.rotaru30@gmail.com

**Keywords:** endodontics, apex locator, sodium hypochlorite, working length

## Abstract

*Background and Objectives*: The aim of this study was to compare the accuracy of three types of electronic apex locators (EALs) when two different concentrations of NaOCl irrigation solutions are used by two operators. *Materials and Methods*: After creating the access cavities for 20 single rooted extracted teeth, the actual canal length (ACL) of each canal was determined visually using a #10 file and magnification. The teeth were subsequently inserted in plastic molds filled with alginate. The electronic measurement of root canal length (EWL) was performed using three different electronic apex locators: Root ZX II, Apex ID, and Dual Pex. Two independent operators, an endodontic specialist with 20 years practice and an undergraduate student in the final year of study, performed the irrigation procedures with two different concentrations of NaOCl (2% and 5.25%), and then measured the EWL using each of the EALs. The accuracy of all EALs, was determined in each case by subtracting the EWL from the ACL. Statistical analyses were performed using one-way ANOVA test. *Results*: In the presence of 2% NaOCl solution, for a margin error of ±0.5 mm, Root ZX II, Apex ID, and Dual Pex presented an accuracy of 90%, 80%, and 85% respectively. The increase in the concentration of the irrigation solution affected the accuracy of Root ZX II and Apex ID for both operators, diminishing it to 75% for the same margin error, but improved Dual Pex’s accuracy to 100%. *Conclusions*: The best accuracy in working length determination was obtained by Root ZX II for 2% NaOCl solution and by Dual Pex for 5.25% NaOCl solution with no significant statistical difference when compared.

## 1. Introduction

Among the factors that significantly influence the success of an endodontic treatment are the correct instrumentation and obturation of root canal to a precise working length (WL). Therefore, an accurate determination of the apical limit during an endodontic treatment is of extreme importance [1]. Different opinions about the ideal end point of root canal instrumentation and obturation were discussed in literature: some authors recommend shaping and filling to the apical foramen (AF) in order to prepare and clean the canal to its whole length [2], while others suggest stopping at the apical constriction (AC) in order to preserve the apical anatomy and avoid damaging the apical tissues [3,4]. What is obvious is that the topography of the constriction seems to have an important influence on the distance between these two references [2,5]. Nevertheless, it is generally accepted that root canal treatment must be carried out within the limits of the root canal [6], especially because the distance between AF and AC may vary from one tooth to another. In a small proportion, AF may coincide to AC [3] for some root canals, but the mean distance between AF and AC in the majority of the cases was reported to be of only 0.2 mm by El Ayouti et al. [3], while other studies reported a mean of 0.5–0.6 mm between the two ending marks of root canals [7]. The extrusion of medication or filling materials, as well as instrumentation beyond the apical terminus, damages the periapical tissues, leading to a delay in tissue healing or causing treatment failure [8]. Furthermore, root canal obturation at 0–2 mm distance from the apical terminus significantly increases the success rate of primary root canal treatments [9].

Methods of determining the working length (WL) include radiological and electronical means. Conventional periapical radiograph was the most used method for determining the length of the root canal. Using this method, the position of the apical AC was chosen according to the radiographic apex. Several microscopic studies have shown that the vast majority of ACs were found between 0.5 and 1 mm from the radiographic apex [10]. Although, because conventional periapical X-ray provides a two-dimensional image of a three-dimensional structure, the accuracy of this procedure is associated with inherent limitations, e.g., distortions, superposition of anatomical structures, image magnification, and subjectivity [11]. Besides, the AF is not always located at the radiological apex, being deviated towards the lingual or buccal side [12]. Its localization by radiological examination is difficult to achieve due to the superimposition over the root structure [13]. These deficiencies of the radiographic WL determination can be eliminated using the electronic method or by combining the two of them.

Even though the idea of using electricity to locate the canal terminus appeared in the beginning of the twentieth century (Cluster 1918, Suzuki 1942), the production of these electronic apex locators (EALs) began only after Sunada (1962) built the first device that electronically determined the WL. The accuracy of early first and second generation EALs was strongly influenced by the presence of strong electrolytes, excessive hemorrhage, or remnant pulp tissue in the root canal [14]. The third generation of EALs used the impedance measurement ratio for two frequencies of the electric power, one high and one low, which were influenced by the change in electrical capacitance at the AF. The values obtained were sent to a microprocessor that processed them mathematically, according to algorithms, thus allowing the localization of the AF. This concept of impedance ratio enabled the development of the Root ZX apex locator (J Morita Corp, Osaka, Japan) by Kobayashi and Suda in 1994 [15]. Due to its exceptional accuracy, demonstrated in numerous investigations, it is still considered the gold standard for EALs [16]. Root ZX II is a self-calibrating EAL, which works on the same principle as its predecessor, calculating impedance ratio for 0.4 and 8 kHz frequencies. Previous studies have shown that Root ZX II was able to determine with great accuracy the AF in the presence of vital or necrotic tissues as well as of various irrigants [17]. The novelty of the fourth generation of EALs was their capacity to measure separately the resistance and the capacitance for two different electric power frequencies. One of them is Apex ID (SybronEndo, Orange, CA, USA), which operates on the same principle as Root ZX II (for 0.5 and 5 kHz), localizing the AF with the same accuracy as this later one [18,19]. Another recently introduced EAL is Dual Pex (Micro Méga, Boissy St-Léger, France), which works accurately in wet and dry root canals, using a multi-frequency technology. However, no data from in vitro or in vivo studies about its accuracy in determining the EWL are currently available in the literature.

Since irrigation is of essential importance in root canal treatment for the removal of dentine, remnant tissues, and individual bacteria or microbial communities [20,21], the determination of WL is often carried out in the presence of various irrigation solutions [22,23]. However, some previous studies [24,25,26] demonstrated that the electroconductive properties of the irrigation solution can influence the accuracy of the EALs leading to shorter or extended measurements. The use of EALs is widespread in dental offices but also in dental schools. Dental students are taught to use them, but the influence of the operator experience on EALs accuracy was poorly evaluated in the literature [27].

Therefore, the aim of this in vitro study was to compare the accuracy of three types of EALs when two different concentrations of NaOCl irrigation solutions are used by operators with different experience in using these devices. The null hypothesis tested was that there are no differences in accuracy among the three EALs tested, regardless of the concentration of the irrigation solution used and operator’s experience.

## 2. Materials and Methods

### 2.1. Tooth Selection

This study was performed after ethical approval was obtained from the Ethics Committee of the “Iuliu Hațieganu” University of Medicine and Pharmacy, Cluj-Napoca, Romania (approval no. 189/30.06.2022).

A number of 20 single rooted teeth (incisors and premolars), extracted due to periodontal or orthodontic reasons, were used in the present study. After extraction, all teeth were immersed in 2.5% NaOCl solution for 4 h (Cloraxid, Cerkamed Stalowa Wola, Poland) in order to disinfect them and allow the disintegration of remained organic tissues. Then they were cleaned from calculus with hand or ultrasonic scalers and stored in saline solution until their use. Before their preparation, all teeth were X-rayed from a buco-oral and mesio-distal incidence, to ensure the presence of a single root canal with a mature apex, without internal or external root resorption and to observe root canal’s degree of curvature. Only teeth with moderate degree of curvature (less than 20°) according to Schneider’s method of evaluation were chosen [28]. All inclusion and exclusion criteria for the selection of the teeth are presented in Table 1.

### 2.2. Sample Preparation

The teeth were numbered from 1 to 20 and cut 4 mm from the cemento-enamel junction in coronal direction with a diamond disc 345 22 MM 0.25 (Yeti dental, Engen, Germany) fitted to a dental hand piece to provide a flat surface and a reliable occlusal reference for all working length (WL) measurements [29]. After creating the access cavities using round diamond burs and Endo Access Burs (Dentsply Sirona, Ballaigues, Switzerland) the coronal thirds of the teeth were shaped with One Flare instrument (Micro Méga, Boissy St-Léger, France) by a single operator and root canals were irrigated with 1 mL 2% NaOCl. Root canal patency was then verified using a size 08 MMC file (Micro Méga, Boissy St-Léger, France).

The ACL of each canal was determined by the insertion of an MMC file #10 (Micro Méga, Boissy St-Léger, France) into the root canal, until its tip could be visualized tangential to the AF under 8× magnification microscope (Alltion AM-6000, Wuzhou, China). A rubber stop was carefully placed at the reference point, represented by the flat edge of the cut, and fixed to the file with polymerized fluid resin. The file was then removed from the canal and the distance between the rubber stop and the tip was measured with an endodontic ruler under endodontic microscope obtaining the actual canal length (ACL). This process was repeated twice by the same operator for each tooth and the measurements recorded in a table. If the AF was located sideways on the surface of the root, the coronal edge of the foramen was taken as a reference for positioning the tip of the file.

The teeth were subsequently inserted separately in plastic molds filled with freshly prepared alginate (Phase, Zhermack SpA, Badia Polesine, Italy) up to the cemento-enamel junction [19,27,30]. The labial electrode of the apex locator was inserted into the alginate as well. The alginate material was prepared according to the manufacturer’s instructions.

### 2.3. Electronic Working Length Determination

The electronic measurements of root canals length were performed using 3 different electronic apex locators:

(a) Root ZX II (J Morita Corp, Osaka, Japan):
(b) Apex ID (SybronEndo, Orange, CA, USA):
(c) Dual Pex (Micro Méga, Boissy St-Léger, France).

For the irrigation protocol, two solutions of 2% NaOCl (S1) and 5.25% NaOCl (S2) were used at room temperature (Chloraxid, Cerkamed Stalowa Wola, Poland). The bottles were opened just before use. Two independent operators, an operator with 20 years of experience in endodontics (Op1) and an undergraduate student (Op2) in the last year of study performed the irrigation procedures and the electronic measurements of the WL with each of the 3 devices. The undergraduate student knew the working principle of EALs and, before starting the research, was trained on the working method of each EAL used in the present study and measured the WL of ten root canals with each of those devices.

In order to ensure an adequate alginate humidity, all measurements were carried out within a 2-h interval [30,31]. Prior to the experiment, all root canals were irrigated with 1 mL saline solution and dried using paper points adapted to root canal.

Root canals were initially irrigated with S1 and then with S2 solution, following the same protocol. Every root canal was first irrigated with 1 mL of S1 solution using a 5 mL syringe and 30-gauge needle (Cerkamed, Stalowa Wola, Poland). The excess irrigation solution from the pulp chamber was removed by air drying, keeping the root canal moist. The external tooth surface was wiped with a cotton pellet. For Root ZX II, the 10 MMC file was connected to the holder and advanced into the root canal until the last green bar appeared on the device display, and “Apex” began to flash. For Apex ID and Dual Pex, the 10 MMC file was advanced into the root canal until the flashing bar “00” was reached. If the instrument remained in this position for at least 5 s, the measurement was considered appropriate. A rubber stop was carefully placed at the reference point of each sample and fixed on the file with light-curing fluid resin. Then, the distance between the rubber stop and the tip of the instrument was measured with an endodontic ruler under dental loupe with 2.5× magnification. After recording the EWL, the irrigation solution (S1) was sucked out and the canal was dried with paper points, adapted to root canal, to avoid the modification of NaOCl concentration [24].

The same protocol was repeated for S2 solution by both operators. Two measurements were carried out for every tooth with each solution (S1 and S2), for all three devices and by both operators. The EALs were used according to the indications given by the manufacturer.

After performing the measurements according to the steps described above, all data were recorded in Excel tables. These EWL measurements were compared with ACL measurements, obtained before the irrigation procedures under the endodontic microscope. The accuracy of EAL was determined in each case by subtracting the EWL from the ACL. Positive or negative values were recorded when the length measured with EALs was lower (the tip was short of the apical foramen) or higher (the tip exceeded the AF) than the one measured by visual detection of the tip under the microscope. When the EALs measurement coincided with the actual length, the value was reported as “0”. All measurement errors were calculated as the absolute difference, in millimeters.

### 2.4. Statistical Analysis

Prior to statistical analyses, a sample size of 20 per group was calculated for one-way ANOVA using G3*Power (software version 3.1.9.6, Erdfelder, Faul and Buchner, Heinrich Heine University, Düsseldorf, Germany), considering alpha-error = 0.05 and power = 0.8.

The normality of statistical distributions was visually assessed using Q-Q plots. One-way ANOVA was applied to determine the accuracy of each EAL in relation with the AF and to assess the influence of operator experience and NaOCl concentration on each EAL separately. Levene’s test was used to establish the homogeneity of variance. With one exception (Op1 vs. Op2 for NaOCl = 5.25%), the equality of variance assumption was not rejected (*p* < 0.05). Pairwise comparisons between groups were assessed using post hoc standard and Dunn tests with Bonferroni corrections. α = 0.05 was chosen as level of statistical significance. All graphics were prepared and analyses performed using JASP (JASP Team 2021, JASP Version 0.16).

## 3. Results

Means and standard deviations of the values obtained, after calculating the differences among ACL and EWL readings obtained by Op1 and Op2 for each EAL and for the two concentrations of NaOCl solutions, are presented in Table 2.

When the measurements obtained with the endodontic microscope (ACL) were compared with those obtained by the three EALs, no statistically significant difference was found among them (*p* > 0.05). In the presence of 2% NaOCl solution, Root ZX II obtained the closest EWL mean value to that of ACL (0.29 mm), when compared to the values obtained by the two other EALs. Yet, no statistically significant difference among the means of the three EALs was found. When irrigation of root canals was performed with 2% NaOCl solution, Root ZX II, Apex ID, and Dual Pex managed to locate in 90%, 80%, and 85%, respectively, the major foramen with a margin of error of ±0.5 mm for Op1. For Op2 the accuracy of the three devices was 85% (Root ZX II), 80% (Apex ID), and 90% (Dual Pex), respectively (Table 3).

The increase in concentration of the irrigation solution (from 2% to 5.25%) affected the accuracy of Root ZX II and Apex ID for both operators, diminishing it to 75% for the same margin error of ±0.5 mm. On the other hand, for Dual Pex, the increase in concentration of the irrigation solution improved its accuracy, achieving the closest value to ACL (0.225 for Op1). As for Op2, an increase in concentration of NaOCl solution did not influence the accuracy of Dual Pex (Table 4).

When comparing the accuracy of Dual Pex versus Apex ID and Dual Pex versus Root ZX II, with S2 solution, a statistically significant difference (*p* < 0.05) was observed for Op1. However, when the three devices were compared two by two by applying the Dunn test with Bonferroni corrections, no significant statistical difference was found among them for Op1 (Table 5).

When 2% NaOCl solution was used for root canal irrigation, the proportion of EWL measurements that were beyond the major foramen for Op1 and Op2 together (*n* = 40) was 22.5% for Root ZX II, 12.5% for Apex ID, and 32.5% for Dual Pex. When 5.25% NaOCl solution was employed, the ratio of the measurements beyond the foramen was 57% for Root ZX II, 60% for Apex ID, and 47% for Dual Pex.

**Figure 1:**
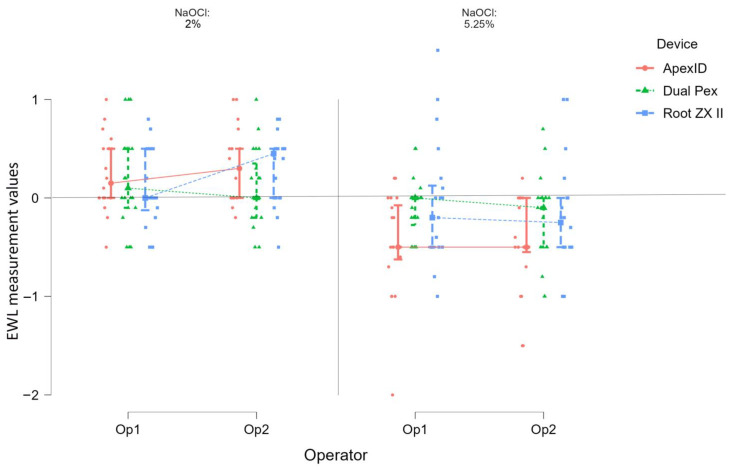
The accuracy of measurements recorded by Apex ID, Dual Pex and Root ZX II for both operators related to ACL (“0”).

The results obtained (*p* > 0.05) showed that the experience of the two operators did not influence the accuracy of the three EALs, regardless of the concentration of the irrigation solution used (Figure 1).

## 4. Discussion

A large number of clinical studies have demonstrated that the outcome of an endodontic treatment depends on the high quality of the root canal instrumentation and three-dimensional filling. Teeth with “flush” root obturation were associated with the highest success rate, while overfilling caused a significant decrease in the healing of periapical tissues [11,32]. The use of electronic devices to determine the distance between the coronal reference point and the AF has increased in recent years because they allow WL determination with greater accuracy than conventional X-rays, thus preventing WL overestimation [33]. However, in more difficult situations, combining the two methods is beneficial for getting the right WL [34].

The null hypothesis tested in the present study was partially accepted because, regardless of the concentration of the irrigation solution used and operator’s experience in irrigating root canals, no significant differences (*p* > 0.05) in the accuracy of the three EALs tested were noticed within a tolerance of ±0.5 mm, except when Op1 used 5.25% NaOCl solution.

For the 2% NaOCl solution, Root ZX II used by Op1 and Dual Pex used by Op2 correctly determined the WL in 90% of cases (within ±0.5 mm tolerance), while Dual Pex used by Op1 and Root ZX II used by Op2 showed an accuracy of 85%. The smallest accuracy in the location of the AF, for this solution, was shown by Apex ID, for both operators (80%). The results obtained by Root ZX II in the present study, when root canals were irrigated with 2% NaOCl solution, are comparable with those of other studies obtained by the same device but with 2.5% NaOCl solution. Therefore, accuracy of 97.44% [29] and 93% [35] was reported for this EAL within a tolerance range of ±0.5 mm, when similar EWL measurement conditions were fulfilled.

Regarding Apex ID accuracy in localizing the AF, the present results do not corroborate with those of Oliviera et al. [35], where this EAL obtained better scores (93% accuracy). Comparable performances for Root ZX (83.33%) and Apex ID (80%) have also been reported when root canals were irrigated with saline solution [36]. As Root ZX II works with the same ratio-method as Root ZX, the results of [36] can be compared with those of the present study.

Since NaOCl solution has electrical conductivity properties, the question arises whether its concentration influences the efficiency of EALs. The present findings revealed that for 5.25% NaOCl irrigation solutions, the accuracy of Root ZX II and Apex ID decreased from 90% to 75% for Root ZX II and from 80% to 75% for Apex ID (within a tolerance range of ±0.5 mm), irrespective of the device or operator, while Dual Pex used by Op1 showed an accuracy of 100% for the same tolerance limit. Therefore, in this case, there was a statistically significant difference (*p* < 0.05) between the results of Dual Pex used by Op1 and the results obtained with the other two EALs by the same operator. Op2 also obtained better results with Dual Pex (80% for a tolerance range of ±0.5 mm) compared to the two other EALs for the same NaOCl solution but without any statistical significance. These findings are in agreement with those of Mahmoud et al. [18] who compared the accuracy of four electronic apex locators using a 5% NaOCl solution for root canal irrigation. The authors found an accuracy of 71.43% for Root ZX II and 68.57% for Apex ID within a tolerance of ±0.5 mm. Similar results were also obtained by Moscoso et al. [37] using a 4% NaOCl solution. They reported for Dentaport ZX (Root ZX II with Endo Motor incorporated) an accuracy of 82.35% when the tolerance range was ±0.5 mm and of 97.05% when the tolerance range was ±1 mm. However, the results of the present study regarding the possible influence of NaOCl concentration solution on the accuracy of EALs are not in agreement with those of Diemer et al. [24]. They concluded that the increase of the concentration of NaOCl solution to 5% brings the accuracy of Root ZXMini from 85% to 90%. Since this EAL is a multi-frequency measurement device, it works similar to Dual Pex. These better results obtained by Dual Pex and Root ZXMini reveal the positive influence of higher concentrations of NaOCl solution on multi-frequency EALs accuracy.

Previous studies have shown that a possible inaccurate measurement of the EWL could be caused by the bending of the measuring file [38]. In order to eliminate this possible source of error, in the present study, the crown of the teeth was cut 4 mm from the cemento-enamel junction and a straight-line access was created by preparing the coronal root canal third with One Flare. For all EALs, the meter reading “apex” or “00” was selected because this was the indication given by the manufacturer for the detection of the major apical foramen. To ensure the accuracy of the EALs, the confirmation of the measurements was made by advancing the file into the root canal until the audio (warning) and visual signals (first red line) showed that the AF was exceeded. Then, the file was retracted in the coronal direction until “apex” or “00” appeared on the screen of the device. Some studies have suggested that this technique increases the accuracy of EALs [31].

By determining the AF with EALs, the WL can be overestimated [33,38,39,40]. Thus ElAyouti et al. [33] found that the proportion of measurements beyond the AF for Root ZX was of 21%. Duran-Sindreu et al. [39], in a similar study, using the same device and a 2.5% NaOCl solution, demonstrated that the file tip passed beyond the AF in 32.1% (tolerance of ±0.5 mm) and 10.7% (tolerance of ±1 mm) of the investigated cases. Apparently, high conductive irrigation solution favors the location of the file tip beyond the apex in the case of Root ZX [41]. In the present investigation, the frequency and value of EWL overestimation was less using 2% NaOCl (22.5% for Root ZX II, 12.5% for Apex ID and 32.5% for Dual Pex) compared with 5.25% NaOCl (57% for Root ZX II, 60% for Apex ID and 47% for Dual Pex) for both operators. A possible explanation for the great number of measurements beyond the AF could be the morphology and the localization of the major apical foramen of the teeth used in the study. Hence, from the 20 teeth analyzed, 11 (55%) of them presented a lateral position of the AF on root apical surface.

In this regard, Ding et al. [42] showed that in the case of lateral foramina, if the coronal edge of the major foramen is taken as the apical reference, the EWL will be slightly overestimated. This conclusion is also supported by Piasecki et al. [19], who proved that a lateral position of the major foramen affects the accuracy of EALs measurements.

The measurements of this investigation consistently located the AF within ±0.5 mm tolerance for all EALs, except three or four cases when the AF was recorded beyond the major apical foramen with 0.5–1 mm. This indicates that, using these devices, after locating the AF and taking off 0.5 mm from the root canal working length obtained, the canal will not be prepared and filled beyond the AF. Nekoofar et al. [14] claim that the use of this method for determining the WL does not show that the AC has been reached, but that the instrumentation and obturation will be conducted inside the root canal.

Some discussion might also be related to the material used for embedding the teeth when determining the EWL. There are a few methods of EWL determination described in literature where gelatin agar or alginate is used to recreate the clinical conditions needed for this procedure [43]. In the present study, the alginate was chosen because it can simulate the consistency of the periodontal ligament by its electroconductive properties [33], having a higher accuracy when used as embedding media for electronic root canal length determination [44]. Moreover, because the teeth were kept in a moist medium until their use, the possibility of losing their conductivity was eliminated [23,25].

Concerning operator experience in the measurement of EWL, no significant differences in the location of the AF between the two operators were observed, regardless of the device or concentration of the irrigation solution used. The results of this study are consistent with those obtained in several studies [27,30] where the use of EALs according to the manufacture’s indications demonstrates that their efficiency cannot be influenced by operator experience.

## 5. Conclusions

Under the limitations of these experimental conditions, including the usage of alginate models for embedding the studied teeth, the three EALs tested in this study located the position of the AF with a similar accuracy. The best results were obtained with Root ZX II for 2% NaOCl and Dual Pex for 5.25% NaOCl, but without statistically significant difference. The concentration of NaOCl solution did not significantly affect the performance of EALs. Electronic root canal measurements represent an objective technique in determining the WL regardless of operator experience. However, the use of these EALs does not entirely prevent the risk of overestimating the WL. Future investigations are needed to determine if other factors, e.g., heated NaOCl solution, the presence of blood or other types of secretions in the root canal, or the rotary motion of endomotors, could influence the accuracy of EALs in determining the WL.

## Figures and Tables

**Table 1:** Inclusion and exclusion criteria for the selected teeth.

Criteria	Inclusion	Exclusion
Teeth	Complete root formation Canal configuration type I Degree of curvature less than 20°	Previous root canal treatment Presence of cracks Presence of root caries Calcified canals Clinical or radiological signs of internal or external root resorptions

**Table 2:** Mean difference and standard deviation of differences between the values recorded under microscope (ACL) and with each electronic apex locator (EAL) for both operators and two concentrations of NaOCl solutions.

Operator	EAL	Solution	N	* Mean	SD
Op1	Apex ID	2%	20	0.325	0.309
		5.25%	20	0.510	0.477
	Dual Pex	2%	20	0.390	0.332
		5.25%	20	0.225	0.220
	Root ZX II	2%	20	0.290	0.269
		5.25%	20	0.470	0.393
Op2	Apex ID	2%	20	0.345	0.344
		5.25%	20	0.495	0.462
	Dual Pex	2%	20	0.305	0.263
		5.25%	20	0.355	0.341
	Root ZX II	2%	20	0.380	0.261
		5.25%	20	0.475	0.360

* All measurement errors were calculated as the absolute difference, in millimeters.

**Table 3:** Comparison of the accuracy among the 3 EALs for both operators and S1 solution.

S1												
	Root ZX II				Apex ID				Dual Pex			
Between	Op1		Op2		Op1		Op2		Op1		Op2	
	*n* = 20	%	*n* = 20	%	*n* = 20	%	*n* = 20	%	*n* = 20	%	*n* = 20	%
* −0.5 to 0	6	30	3	15	3	15	2	10	6	30	7	35
0	7	35	4	20	6	30	7	35	4	20	4	20
0 to 0.5	5	25	10	50	7	35	7	35	7	35	7	35
0.5 to 1	2	10	3	15	4	20	4	20	3	15	2	10

* Negative value indicates measurements longer than ACL.

**Table 4:** Comparison of the accuracy between EALs for both operators and S2 solution.

S2												
	Root ZX II				Apex ID				Dual Pex			
Between	Op1		Op2		Op1		Op2		Op1		Op2	
	*n* = 20	%	*n* = 20	%	*n* = 20	%	*n* = 20	%	*n* = 20	%	*n* = 20	%
* <−1	0	-	0	-	1	5	2	10	0	-	0	-
* −1 to −0.5	2	10	3	15	3	15	3	15	0	-	3	15
^∗^ −0.5 to 0	9	45	10	50	10	50	9	45	9	45	8	40
0	3	15	3	15	3	15	5	25	7	35	6	30
0 to 0.5	3	15	2	10	2	10	1	5	4	20	2	10
0.5 to 1	2	10	2	10	1	5	0	-	0	-	1	5
<1	1	5	0	-	0	-	0	-	0	-	0	-

* Negative value indicates measurements longer than ACL.

**Table 5:** Comparison of the accuracy between the three EALs for Op1 and S2 (Dunn’s Post Hoc Comparisons with Bonferroni corrections).

EAL	*p*	*p*_bonf_	*p*_holm_
ApexID—Dual Pex	*	0.068	0.068
ApexID—Root ZX II	0.831	1.000	0.831
Dual Pex—Root ZX II	*	0.116	0.077

* *p* < 0.05.

## Data Availability

The data presented in this study are available on request from the authors.
